# C2238/*α*ANP modulates apolipoprotein E through Egr-1/miR199a in vascular smooth muscle cells *in vitro*

**DOI:** 10.1038/cddis.2015.370

**Published:** 2015-12-31

**Authors:** R Stanzione, S Sciarretta, S Marchitti, F Bianchi, S Di Castro, S Scarpino, M Cotugno, G Frati, M Volpe, S Rubattu

**Affiliations:** 1 IRCCS Neuromed, Pozzilli (Is), Sapienza University of Rome, Latina, Italy; 2 Department of Medical-Surgical Sciences and Biotechnologies, Sapienza University of Rome, Latina, Italy; 3 Department of Clinical and Molecular Medicine, School of Medicine and Psychology, Sapienza University of Rome, Ospedale S. Andrea, Rome, Italy

## Abstract

Subjects carrying the T2238C ANP gene variant have a higher risk to suffer a stroke or myocardial infarction. The mechanisms through which T2238C/*α*ANP exerts detrimental vascular effects need to be fully clarified. In the present work we aimed at exploring the impact of C2238/*α*ANP (mutant form) on atherosclerosis-related pathways. As a first step, an atherosclerosis gene expression macroarray analysis was performed in vascular smooth muscle cells (VSMCs) exposed to either T2238/*α*ANP (wild type) or C2238/*α*ANP. The major finding was that apolipoprotein E (*ApoE*) gene expression was significantly downregulated by C2238/*α*ANP and it was upregulated by T2238/*α*ANP. We subsequently found that C2238/*α*ANP induces *ApoE* downregulation through type C natriuretic peptide receptor (NPR-C)-dependent mechanisms involving the upregulation of miR199a-3p and miR199a-5p and the downregulation of DNAJA4. In fact, NPR-C knockdown rescued ApoE level. Upregulation of miR199a by NPR-C was mediated by a reactive oxygen species-dependent increase of the early growth response protein-1 (Egr-1) transcription factor. In fact, Egr-1 knockdown abolished the impact of C2238/*α*ANP on ApoE and miR199a. Of note, downregulation of ApoE by C2238/*α*ANP was associated with a significant increase in inflammation, apoptosis and necrosis that was completely rescued by the exogenous administration of recombinant ApoE. In conclusion, our study dissected a novel mechanism of vascular damage exerted by C2238/*α*ANP that is mediated by ApoE downregulation. We provide the first demonstration that C2238/*α*ANP downregulates ApoE in VSMCs through NPR-C-dependent activation of Egr-1 and the consequent upregulation of miR199a. Restoring ApoE levels could represent a potential therapeutic strategy to counteract the harmful effects of C2238/*α*ANP.

Atrial natriuretic peptide (ANP) plays important cytoprotective cardiovascular functions, such as antihypertrophic, antiproliferative and prosurvival effects in cardiomyocytes, vascular smooth muscle cells (VSMCs), fibroblasts and endothelial cells.^1^ Recent work demonstrated that a molecular variant of *α*ANP (C2238/*α*ANP), which is characterized by the C-terminal extension of the peptide with two arginine residues and is secondary to the substitution of a thymine for a cytosine at position 2238 of the *ANP* gene, exerts detrimental vascular effects. In fact, we and other groups previously showed that subjects carrying the T2238C ANP gene variant have a significantly higher risk to have a stroke or a myocardial infarction and display a significant increase of circulating markers of leukocyte activation.^2, 3, 4, 5, 6, 7, 8^ Overall, this evidence highlights the need to find therapeutic targets to prevent or reduce the detrimental effects of C2238/*α*ANP gene variant, which is relatively frequent in the general population. However, this is possible only if the molecular mechanisms through which C2238/*α*ANP favors the development of vascular dysfunction, atherosclerosis and cardiovascular accidents are fully elucidated.

We previously reported that C2238/*α*ANP molecular variant promotes cellular apoptosis, necrosis and oxidative stress, whereas it reduces cell proliferation, angiogenesis and vasodilation.^9, 10, 11^ Mechanistically, we found that the deleterious effects of C2238/*α*ANP variant are mediated by a deregulated activation of the type C natriuretic peptide receptor (NPR-C) with a consequent decrease of cAMP levels and PKA activity.^10, 11^ The abnormal activation of NPR-C was found to be associated with a reduction of the Akt/eNOS pathway and an increase in NADPH oxidase-derived reactive oxygen species (ROS) activity in endothelial cells.^9, 10^ In addition, an epigenetic phenomenon involving miR21 significantly contributed to the detrimental effects of C2238C/*α*ANP on cell viability and function in VSMCs.^11^ However, these molecular mechanisms can only explain the deleterious properties of C2238/*α*ANP on atherosclerotic plaque stability. On the other hand, the interaction of C2238/*α*ANP with multiple genes/proteins directly related to the atherosclerotic process remains unknown. This latter possibility is supported by the known involvement of natriuretic peptides in atherosclerosis.^1^

For this reason, we conducted a gene expression macroarray analysis in VSMCs, a key cellular element in atherosclerosis development^12^ and a target of ANP,^1^ in order to investigate the potential impact of T2238C/*α*ANP on mechanisms underlying atherosclerosis. We found that C2238/*α*ANP induces a significant downregulation of the apolipoprotein E (*ApoE*) gene expression, encoding a known antiatherosclerotic and anti-inflammatory protein, through the activation of the NPR-C/Egr-1/miR199a/DNAJA4 signaling pathway that we dissect here for the first time. ApoE downregulation was found to be responsible for the detrimental cellular abnormalities induced by T2238C/*α*ANP in VSMCs.

## Results

### Differential *ApoE* gene expression in VSMCs induced by TT2238/*α*ANP and CC2238/*α*ANP

**Figure 1:**
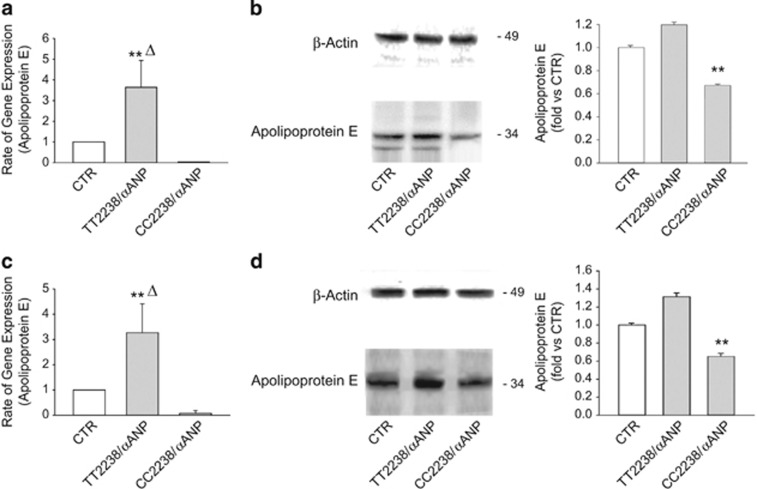
Differential regulation of ApoE mRNA and protein levels by TT2238/*α*ANP and CC2238/*α*ANP in HUVSMCs and CAMSCs. Results of RT-PCR (**a** and **c**) and of western blotting (**b** and **d**, with corresponding densitometric analysis) for ApoE expression levels in HUVSMCs (upper part) and in CASMCs (lower part). Results are expressed as mean±S.D. Number of independent experiments=6. Δ *P*<0.005 for TT2238/*α*ANP *versus* CTR. **P<0.0001 for TT2238/*α*ANP *versus* CC2238/*α*ANP (**a** and **b**) and for CC2238/*α*ANP *versus* both CTR and TT2238/*α*ANP (**b** and **d**). CC2238/*α*ANP, variant *α*ANP; TT2238/*α*ANP, wild-type *α*ANP; CTR, control

Atherosclerosis genes expression profile of human umbilical vein smooth muscle cells (HUVSMCs) exposed to either TT2238/*α*ANP or CC2238/*α*ANP, compared with unstimulated cells, is reported in Table 1. Among the sequences that were significantly and differentially regulated (at least 5-fold) by the two *α*ANP forms, ApoE gene was significantly reduced by CC2238/*α*ANP, whereas it was markedly stimulated by TT2238/*α*ANP. We validated the macroarray results by demonstrating that both ApoE mRNA and protein expression levels were downregulated in both HUVSMCs (Figures 1a and b) and coronary artery SMCs (CAMSCs) (Figures 1c and d) by CC2238/*α*ANP, whereas they were upregulated by TT2238/*α*ANP. Since it is well known that ApoE exerts critical antiatherosclerotic and anti-inflammatory actions,^13^ we decided to evaluate whether ApoE downregulation mediates the detrimental effects of CC2238/*α*ANP.

### CC2238/*α*ANP induces ApoE downregulation through NPR-C-dependent mechanisms associated with the upregulation of miR199a

**Figure 2:**
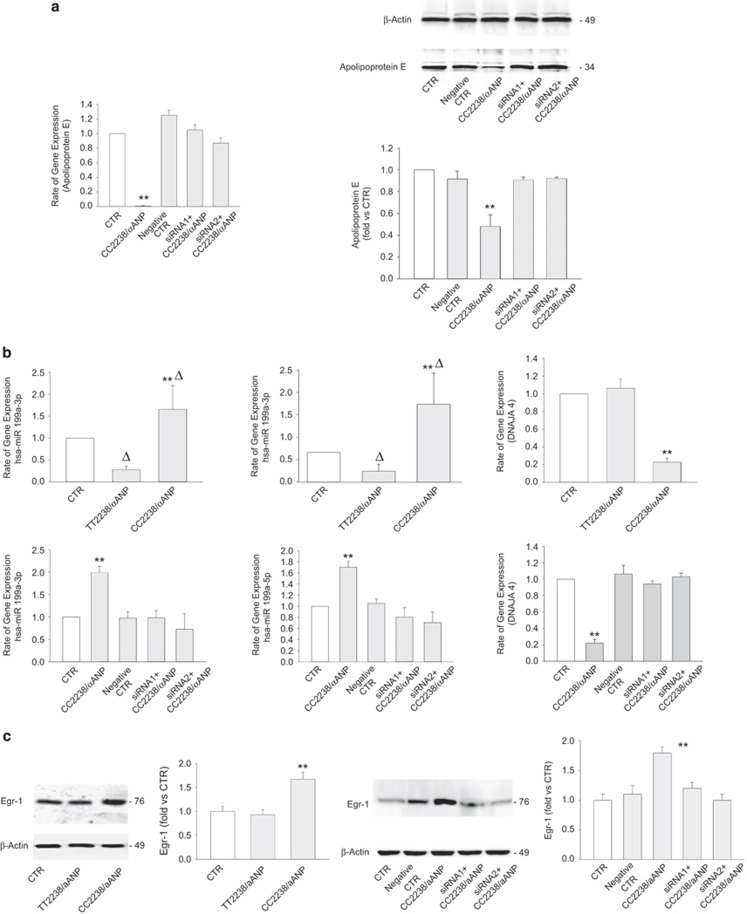
C2238/*α*ANP induces ApoE downregulation through NPR-C-dependent mechanisms associated with the upregulation of miR199a and of Egr-1 in HUVSMCs. (**a**) Analysis by RT-PCR and by western blotting of ApoE expression levels upon CC2238/*α*ANP in NPR-C not silenced and silenced cells. **P<0.0001 for CC2238/*α*ANP *versus* all other samples. (**b**) miR199a-3p, miR199a-5p, DNAJA4 mRNA levels under either TT2238/*α*ANP or CC2238/*α*ANP (upper part of the panel) and in NPR-C not silenced and silenced cells under CC2238/*α*ANP (lower part of the panel). Two specific siRNAs were used to obtain NPR-C gene silencing. **P<0.0001 for CC2238/*α*ANP *versus* TT2238/*α*ANP and CTR. Δ*P*<0.005 for TT2238/*α*ANP *versus* CTR and for CC2238/*α*ANP *versus* CTR (miR199a-3p and miR199a-5p). (**c**) Representative western blotting of Egr-1, with corresponding densitometric analysis under either TT2238/*α*ANP or CC2238/*α*ANP exposure (left side) and under CC2238/*α*ANP in NPR-C not silenced and silenced cells (right side). **P<0.0001 for CC2238/*α*ANP *versus* all other samples. Results are expressed as mean±S.D. Number of independent experiments=6. CC2238/*α*ANP, variant *α*ANP; TT2238/*α*ANP, wild-type *α*ANP; siRNA1, NPR-C gene silencer 1; siRNA2, NPR-C gene silencer 2; hsa-miR199a, human microRNA199a; Egr-1, early growth response protein-1; CTR, control

**Figure 3:**
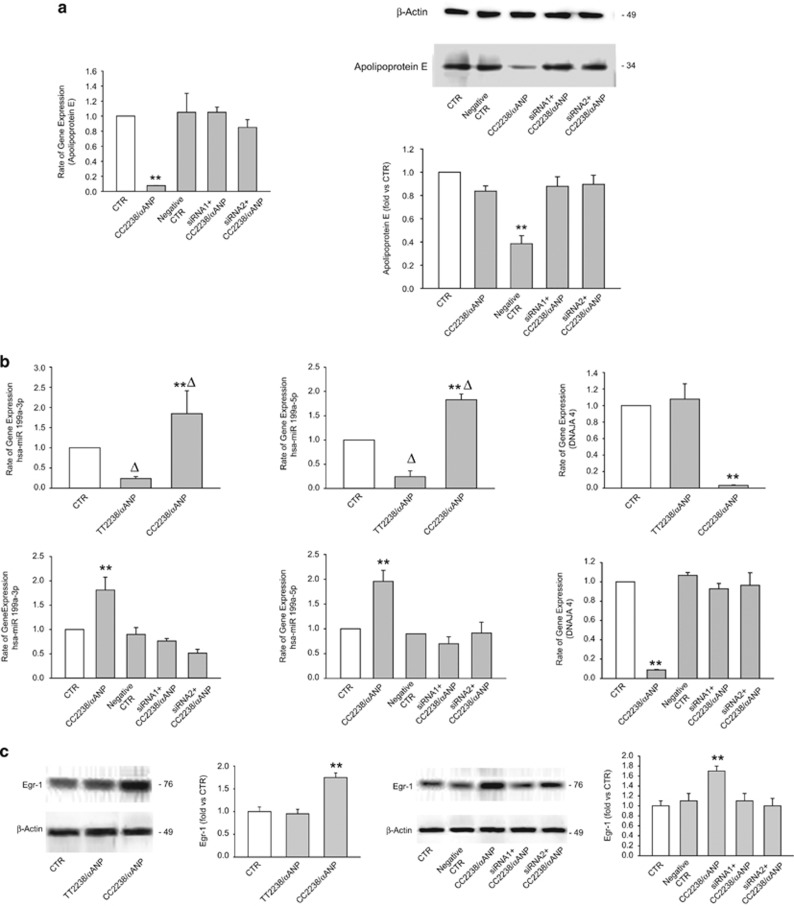
C2238/*α*ANP induces ApoE downregulation through NPR-C-dependent mechanisms associated with the upregulation of miR199a and of Egr-1 in CASMCs. (**a**) Analysis by RT-PCR and by western blotting of ApoE expression levels upon CC2238/*α*ANP in NPR-C not silenced and silenced cells. **P<0.0001 for CC2238/*α*ANP *versus* all other samples. (**b**) miR199a-3p, miR199a-5p, DNAJA4 mRNA levels under either TT2238/*α*ANP or CC2238/*α*ANP (upper part of the panel) and in NPR-C not silenced and silenced cells under CC2238/*α*ANP (lower part of the panel). Two specific siRNAs were used to obtain NPR-C gene silencing. **P<0.0001 for CC2238/*α*ANP *versus* TT2238/*α*ANP and CTR. Δ*P*<0.005 for TT2238/*α*ANP *versus* CTR and for CC2238/*α*ANP *versus* CTR (miR199a-3p and miR199a-5p). (**c**) Representative western blotting of Egr-1, with corresponding densitometric analysis under either TT2238/*α*ANP or CC2238/*α*ANP exposure (left side) and under CC2238/*α*ANP in NPR-C not silenced and silenced cells (right side). **P<0.0001 for CC2238/*α*ANP *versus* all other samples. Results are expressed as mean±S.D. Number of independent experiments=6. For abbreviations see Figure 2

We then dissected the mechanisms through which CC2238/*α*ANP induces the reduction of ApoE expression. Previous work from our laboratory demonstrated that CC2238/*α*ANP induces detrimental vascular effects through a deregulated activation of NPR-C.^10, 11^ Therefore, we investigated whether NPR-C mediates the effects of CC2238/*α*ANP on ApoE expression levels. Our results confirmed this hypothesis, since we found that NPR-C knockdown rescued ApoE expression levels in both HUVSMCs and coronary artery smooth muscle cells (CASMCs) exposed to CC2238/*α*ANP (Figures 2a and 3a).

Of note, previous evidence demonstrated that ApoE expression levels are epigenetically regulated by miR199a-3p and miR199a-5p, which in turn can either directly target ApoE or target the heat-shock protein DNAJA4, which promotes the upregulation of ApoE.^14^ In line with this evidence, we found that miR199a-3p and miR199a-5p expression levels are significantly upregulated by CC2238/*α*ANP, whereas they are downregulated by TT2238/*α*ANP. On the other hand, the DNAJA4 expression level resulted to be downregulated by CC2238/*α*ANP, whereas it was unchanged in cells exposed to TT2238/*α*ANP (Figures 2b and 3b). Interestingly, we observed that NPR-C knockdown rescued miR199a-3p, miR199a-5p and DNAJA4 expression levels in both VSMC lines treated with CC2238/*α*ANP (Figures 2b and 3b). These data suggest that NPR-C regulates ApoE levels through the regulation of miR199a-3p, miR199a-5p and DNAJA4.

### NPR-C regulates miR199a through the ROS-dependent activation of early growth response protein-1 (Egr-1)

**Figure 4:**
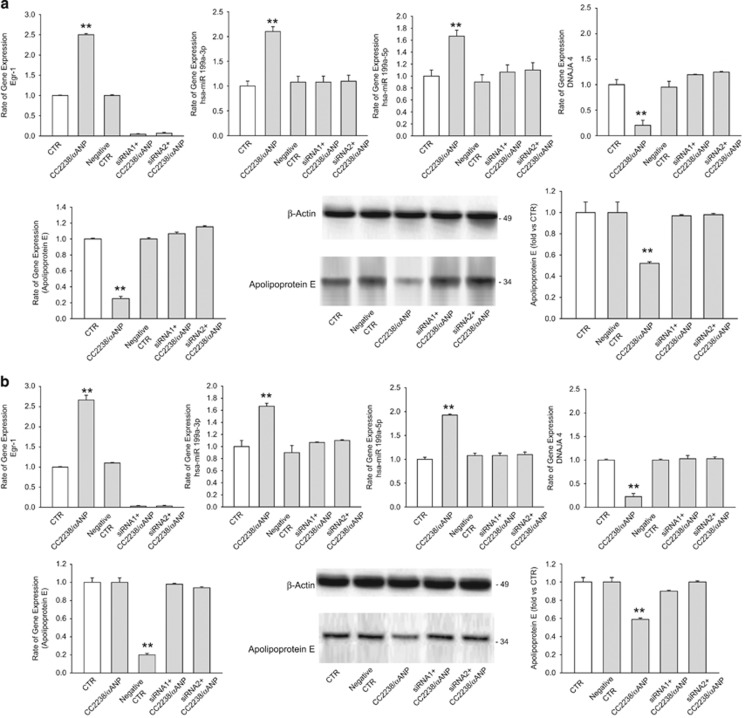
Impact of Egr-1 gene silencing on the ApoE regulatory pathway in both HUVSMCs and CAMSCs. (**a**) Impact of Egr-1 gene silencing on Egr-1, miR199-3p, miR199-5p, DNAJA4 expression levels (as detected by RT-PCR) and on ApoE expression levels (as detected by both RT-PCR and western blotting) in HUVSMCs. Two specific siRNAs were used to obtain Egr-1 gene disruption. (**b**) Impact of Egr-1 gene silencing on Egr-1, miR199-3p, miR199-5p, DNAJA4 expression levels (as detected by RT-PCR), and on ApoE expression levels (as detected by both RT-PCR and western blot) in CASMCs. Results are expressed as mean±S.D. Number of independent experiments=4 for each cell lines. **P<0.0001 for CC2238/*α*ANP *versus* all other points. siRNA1, Egr-1 gene silencer 1; siRNA2, Egr-1 gene silencer 2; for other abbreviations see Figures 2 and 3

Then, we tried to elucidate the signaling mechanisms through which NPR-C promotes the upregulation of miR199a-3p and miR199a-5p, the downregulation of DNAJA4 and ultimately the downregulation of ApoE expression levels in response to CC2238/*α*ANP. The transcription factor Egr-1 was previously found to promote the upregulation of miR199a.^15^ In addition, Egr-1 is often found activated in the vasculature during stress and was described to be involved in the atherosclerotic process.^16^ Therefore, we evaluated whether Egr-1 is involved in the NPR-C-dependent regulation of miR199a-3p and miR199a-5p in response to CC2238/*α*ANP. We found that Egr-1 is upregulated in response to CC2238/*α*ANP and this effect was abrogated by NPR-C knockdown (Figures 2c and 3c). CC2238/*α*ANP is known to induce NPR-C-dependent NADPH oxidase activation and ROS production.^9, 10^ Egr-1 can be activated by ROS.^17^ In accordance, we found that C2238/*α*ANP-dependent upregulation of Egr-1 was blunted by apocynin, a NADPH oxidase inhibitor (Supplementary Figure S1). Finally, we found that Egr-1 knockdown normalized miR199a and DNAJA4 levels and blocked the ApoE downregulation in both VSMC lines treated with CC2238/*α*ANP (Figure 4). Overall, these data indicate that CC2238/*α*ANP leads to miR199a upregulation and ApoE downregulation in VSMCs through the NPR-C-dependent ROS production which in turn leads to Egr-1 activation.

### CC2238/*α*ANP modulates apoptosis, necrosis and inflammation in VSMCs through NPR-C-dependent ApoE downregulation

Then, we evaluated the biological significance of ApoE downregulation in cells exposed to CC2238/*α*ANP. Supplementary Figures S2 and S3 show the results of western blotting analysis to evaluate the expression levels of cleaved caspase-3, cellular repressor of E1A-stimulated gene (CREG), and both phosphorylated and total forms of JNK and p38MAPK in HUVSMCs (Supplementary Figure S2) and in CASMCs (Supplementary Figure S3) in the presence of CC2238/*α*ANP. Cleaved caspase-3 was increased by CC2238/*α*ANP (Supplementary Figures S2A and S3A). On the other hand, expression of CREG, an antiapoptotic factor, was reduced (Supplementary Figures S2B and S3B). Consistently with previous studies showing that CREG inhibits VSMC apoptosis through the inhibition of p38MAPK and JNK signaling pathways,^18^ we observed a parallel increased phosphorylation of both p38MAPK and JNK in both HUVSMCs and CASMCs treated with CC2238/*α*ANP as compared with control cells (Supplementary Figures S2C, D, S3C and D). Of note, NPR-C knockdown completely rescued the CC2238/*α*ANP effects on cleaved-caspase-3, phospho-p38MAPK, phosphoJNK and CREG. Cellular levels of NF-*κ*B and Smad4, known markers of inflammation, were also significantly increased in both HUVSMCs and CASMCs exposed to CC2238/*α*ANP (Supplementary Figures S2E, F, S3E and F). NPR-C gene silencing blunted the increase of both NF-*κ*B and Smad4 despite the presence of CC2238/*α*ANP in both VSMC lines (Supplementary Figures S2E, F, S3E and F). Altogether, these findings indicate that C2238/*α*ANP induces apoptosis and inflammation in VSMCs through NPR-C-dependent mechanisms.

**Figure 5:**
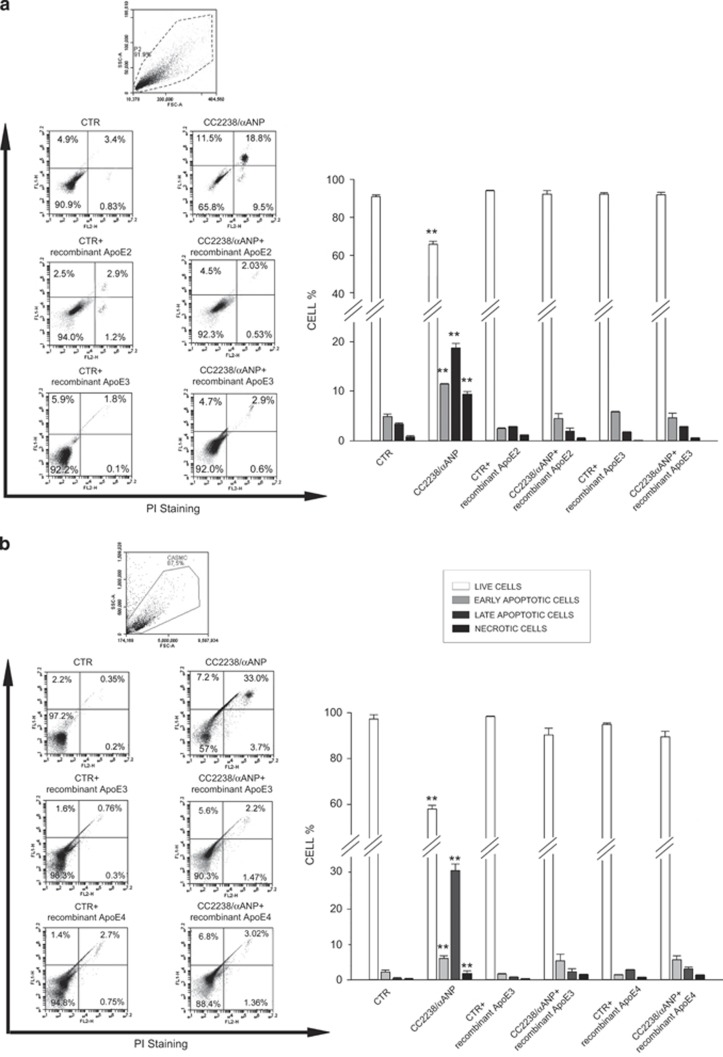
Effect of recombinant ApoE on cell viability in the presence of CC2238/*α*ANP in both HUVSMCs (**a**) and in CASMCs (**b**) Cells were concomitantly exposed to recombinant ApoE and CC2238/*α*ANP. At the end of stimulation, cell viability was analyzed by FACS. Each figure panel shows representative scatter plots for each experimental condition (left side) and corresponding densitometric analysis (right side). Results are expressed as mean±S.D. Number of independent experiments=3 for each ApoE isoform. **P<0.0001 for CC2238/*α*ANP *versus* all other points. CC2238/*α*ANP, variant *α*ANP; CTR, control; PI, propidium iodide; ApoE, apolipoprotein E; CTR, control

**Figure 6:**
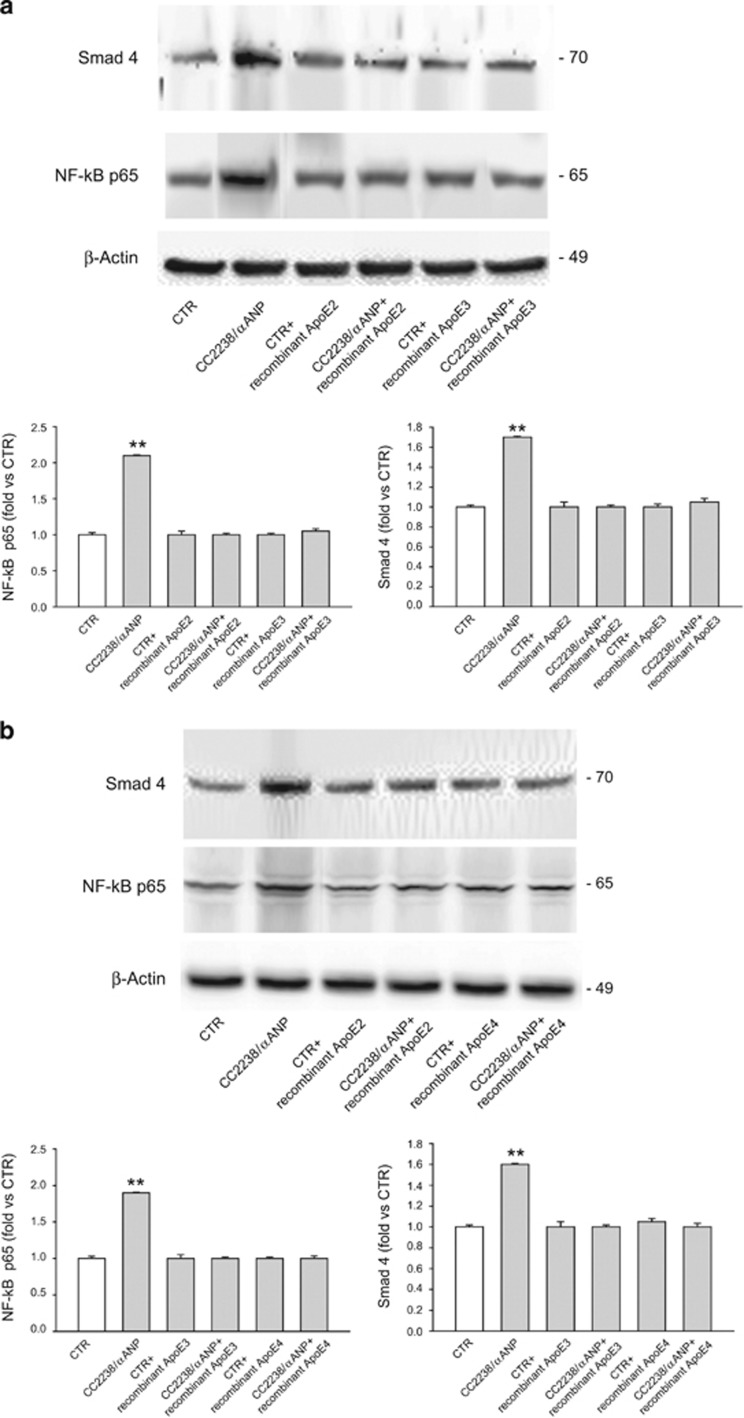
Effect of recombinant ApoE on markers of inflammation in the presence of CC2238/*α*ANP in both HUVSMCs (**a**) and CASMCs (**b**). Cells were concomitantly exposed to recombinant ApoE and CC2238/*α*ANP. Twenty-four hours after completion of stimulation, total proteins were analyzed for markers of inflammation by western blot. (**a**) Representative western blot of Nf-*κ*B and Smad4, with corresponding densitometric analysis, in HUVSMCs. (**b**) Representative western blot of Nf-*κ*B and Smad4, with corresponding densitometric analysis, in CASMCs. Data are expressed as mean±S.D. Number of independent experiments=3 for each ApoE isoform. **P<0.0001 for CC2238/*α*ANP *versus* all other points. CC2238/*α*ANP, variant *α*ANP; ApoE, apolipoprotein E; CTR, control

Of note, we found that these mechanisms are mediated by ApoE downregulation. First of all, sequencing analysis revealed that HUVSMCs express the ApoE isoforms 2 and 3 (APOE2/APOE3) whereas CASMCs express ApoE isoforms 3 and 4 (APOE3/APOE4) (Supplementary Figure 4). More importantly, we found that restoration of ApoE levels in both HUVSMCs (Figure 5a) and CASMCs (Figure 5b) treated with CC2238/*α*ANP through the concomitant exogenous administration of the corresponding ApoE recombinant isoform was able to rescue cell viability, which was significantly impaired by CC2238/*α*ANP alone as compared with untreated cells. ApoE administration significantly reduced both early and late apoptosis and necrosis induced by CC2238/*α*ANP. In addition, VSMCs showed a significant decrease of inflammation under exposure to recombinant ApoE despite the presence of CC2238/*α*ANP (Figure 6).

## Discussion

**Figure 7:**
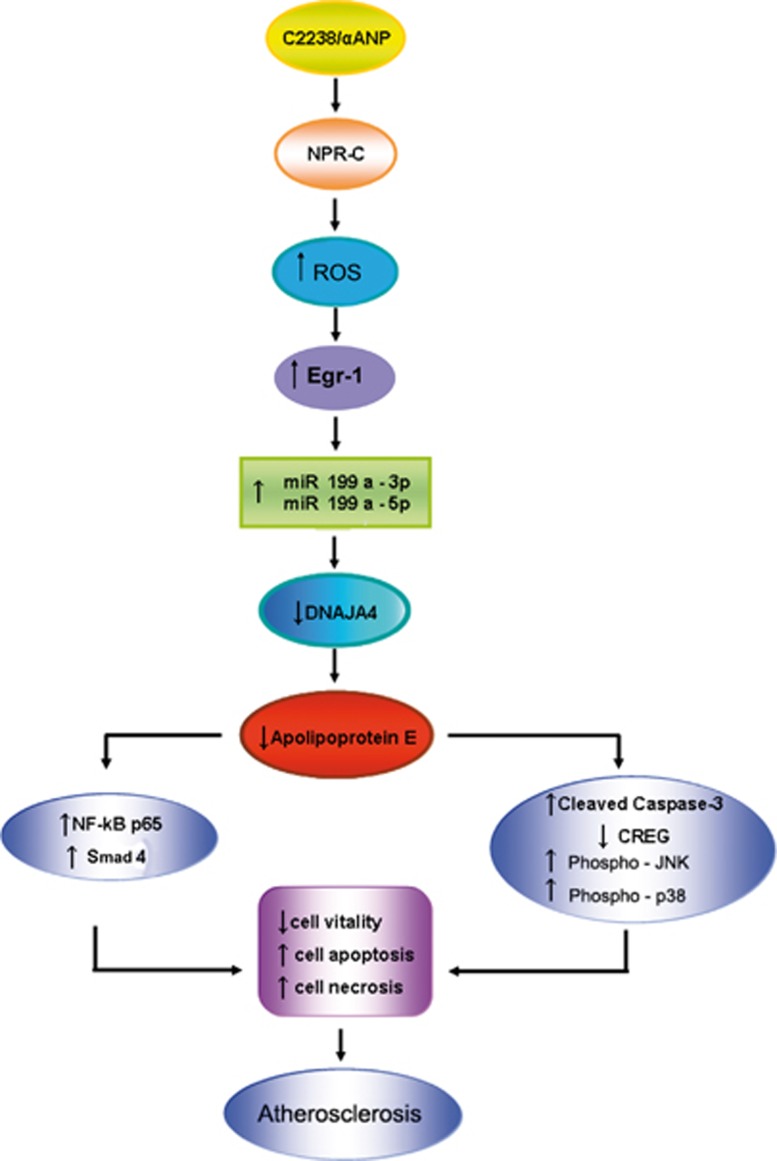
Schematic representation of the pathway involving NPR-C, Egr-1, miR199a-3p and miR199a-5p on ApoE modulation upon CC2238/*α*ANP exposure in VSMCs. The positive effects of ApoE supplementation to VSMCs, despite CC2238/*α*ANP exposure, support the strong contribution of this pathway into promotion of vascular damage through the final ApoE downregulation. C2238/*α*ANP, variant *α*ANP; NPR-C, type C natriuretic peptide receptor; ROS, reactive oxygen species; Egr-1, early growth response protein-1; miR199a, microRNA199a; CREG, cellular repressor of E1A-stimulated gene; JNK, c-Jun N-terminal kinase

T2238C/*α*ANP gene variant is an emerging non-modifiable cardiovascular risk factor. In fact, subjects carrying this genetic variant have a higher risk for occurrence of cardiovascular adverse events, such as stroke and myocardial infarction. In addition, these subjects display signs of endothelial dysfunction already at a young age. Here, we analyzed the impact of the T2238C/*α*ANP gene variant on atherosclerosis-related mechanisms. We found that C2238/*α*ANP significantly downregulates *ApoE* gene expression in VSMCs through NPR-C-dependent mechanisms. We also demonstrated that C2238/*α*ANP plays this effect through a selective epigenetic regulation of ApoE involving Egr-1 and two small noncoding RNAs (miR199a-3p and miR199a-5p). Most importantly, we provided evidence that the detrimental effects induced by the C2238/*α*ANP–NPR-C axis in VSMCs, such as increased rate of apoptosis, necrosis and inflammation, could be fully recovered by recombinant ApoE protein supplementation (Figure 7).

These data significantly extend our previous evidence demonstrating that C2238/*α*ANP induces endothelial cell death and oxidative stress, reduces angiogenesis and promotes endothelial dysfunction through the deregulated activation of NPR-C/cAMP signaling and the reduction of the PKA/Akt/eNOS pathway.^9, 10^ In addition, we recently found that C2238/*α*ANP increases oxidative stress and cellular migration and promotes vasoconstriction through the NPR-C/cAMP/PKA/CREB/miR21 signaling pathway.^11^ The current study demonstrates for the first time a direct impact of C2238/*α*ANP on mechanisms underlying atherosclerosis in VSMCs, a key element in this process. In fact, C2238/*α*ANP induces a significant downregulation of ApoE which plays an important role on atherosclerosis development.^19, 20^ ApoE is a 34-kDa protein that participates in the mobilization and distribution of cholesterol and of other lipids among various tissues of the body.^20^ It is known that ApoE deficiency alone promotes the development of aortic atherosclerotic plaques in mice.^21^ Previous studies demonstrated that ApoE exerts also pleiotropic antiatherosclerotic and anti-inflammatory cellular effects. ApoE was previously found to be expressed in VSMCs where ApoE downregulation is positively correlated with typical markers of inflammation such as Smad4^22^ and NF-*κ*B.^23^ In addition, ApoE hampers the proinflammatory functions of inflammatory cells.^24^ In humans, where lack of ApoE is rare, risk of atherosclerosis is strongly associated with three common apoE isoforms in the order of APOE4>APOE3>APOE2.^20^ Furthermore, ApoE is known to play a role in the development of both cardiovascular^25^ and neurological diseases.^26^ Therefore, our study may suggest that C2238/*α*ANP induces cellular abnormalities through the inhibition of the above-described pleiotropic effects of ApoE. Furthermore, since higher myeloperoxidase levels were reported in Apo^−/−^ mice,^27^ we can postulate that C2238/*α*ANP may, at least in part, associate to higher myeloperoxidase levels, as previously reported in coronary artery disease patients,^4^ through its ability to lower ApoE gene expression.

On the other hand, our current results indicate that wild-type *α*ANP may exert physiological antiatherosclerotic effects by upregulating ApoE levels. This possibility is supported by previous evidence suggesting a close relationship between natriuretic peptides (NPs) and atherosclerosis. In fact, NPs are significantly more expressed in human coronary explants of advanced atherosclerotic lesions.^28^ Inhibition of NP degradation by NEP inhibitor candoxatril prevented fatty deposit in a rabbit model.^29^ Circulating NP levels increase in parallel to the increase of coronary plaque stenosis, reaching a plateau level at the highest degree of vessel stenosis.^30^

Notably, previous work has shown that atherosclerosis increases in an animal model lacking both NPR-A and ApoE (Npr1^−/−^ ApoE^−/−^ mice),^31^ suggesting that lack of NPR-A could induce VSMC growth through other receptor signaling, thus enhancing the negative vascular effects due to lack of ApoE.

Another original finding of our study is the demonstration that C2238/*α*ANP induces ApoE downregulation through the NPR-C-dependent upregulation of Egr-1. This effect is mediated by NADPH oxidase activity, since apocynin abrogated the effects of C2238/*α*ANP on Egr-1 activation. To our knowledge, this is the first time that a negative epigenetic regulation of ApoE by Egr-1 is demonstrated in the vascular context. This finding also extends previous evidence advocating a role of Egr-1 activation in the development of atherosclerosis and may suggest that Egr-1 activation may contribute to atherosclerosis through the downregulation of ApoE. Interestingly, previous work demonstrated that atherosclerosis is reduced in a double knockout model of Egr-1 and ApoE mice, as compared with that observed in the ApoE^−/−^ knockout model only, thus suggesting that lack of Egr-1 is protective in the absence of ApoE.^32^ Our study extends this evidence by demonstrating that Egr-1 can be upstream of ApoE, negatively regulating its expression.

Of note, we found that Egr-1 activation by C2238/*α*ANP promotes the upregulation of miR199a-3p and miR199a-5p, thus confirming previous findings demonstrating that Egr-1 regulates miR199a.^15^ These data suggest that Egr-1 may epigenetically regulate ApoE through these two microRNAs (miRNAs). In fact, Pencheva *et al.*^14^ have recently demonstrated that miR199a-3p and miR199a-5p regulate melanoma metastasis formation through the downregulation of ApoE levels. The authors found that these miRNAs may either target ApoE directly or indirectly inhibit ApoE levels through the targeting of DNAJA4, a heat-shock protein stimulating ApoE levels. In line with this evidence, we also found that C2238/*α*ANP/NPR-C/Egr-1/miR199a signaling promotes the downregulation of DNAJA4 levels.

Of note, these findings reinforce available evidence elucidating the role of miRNAs in the pathogenesis of VSMC abnormalities and of vascular diseases in general. In this regard, we previously found that a dysregulation of miR21 contributed to VSMC derangements upon C2238/*α*ANP stimulation.^11^ In addition, downregulation of miR23b in injured vascular wall was recently found to modulate VSMC phenotypic switching through the involvement of both SMAD3 and FOXO4.^33^ Overall, this evidence supports the knowledge that miRNA-dependent epigenetic mechanisms can regulate the pathogenesis of human diseases in a highly integrative manner.

Previous deliveries of human ApoE3 by intramuscular plasmid injection and adenovirus,^34^ by synthetic apolipoprotein mimetic peptides^35^ or by a cell-based platform,^36^ have inhibited the development of atherosclerotic lesions. We first assessed the specific ApoE isoform expressed by the two VSMC lines used in our studies, which was never reported before. Consequently, we observed full benefits from the exposure of VSMCs to the specific recombinant ApoE isoform, despite the presence of C2238/*α*ANP. The pleiotropic effects of ApoE can well explain our results, since this protein is atheroprotective not only through its lipid-lowering effect but also through antioxidant and anti-inflammatory activities.^19^ The beneficial results obtained through ApoE treatment of damaged VSMCs exposed to C2238/*α*ANP highlight the potential key role of this molecule in the treatment of atherosclerosis in subjects with C2238/*α*ANP variant, although several limitations still exist for its clinical application.

In summary, we report the first evidence of a novel vascular disease mechanism in VSMCs mediated by NPR-C, involving Egr-1 and two dependent small noncoding RNAs, ultimately leading to ApoE downregulation, in the presence of the C2238/*α*ANP gene variant, an emerging cardiovascular risk factor. This newly discovered transduction cascade contributes to the wide range of damaging vascular actions due to C2238/*α*ANP that are in contrast with the known beneficial properties of wild-type *α*ANP. Moreover, our novel findings discover unknown pleiotropic actions of NPR-C in vascular cells through its involvement in the epigenetic regulation of vascular antiatherosclerotic proteins, such as apolipoprotein E.

## Materials and Methods

### Stimulation of HUVSMCs with TT2238 and CC2238/*α*ANP and RNA extraction for the atherosclerosis macroarray analysis

Commercially available HUVSMCs were stimulated with either synthetic wild type (TT2238) or mutant (CC2238)/*α*ANP at a final concentration of 10^9^ mol/l for 12 h, as previously described.^9, 10, 11^ Control plates received *α*ANP-free medium. Three experiments were performed. Total RNA, obtained at the end of stimulation, was used for cDNA synthesis and then for the macroarray analysis. Confirmation of ApoE gene and protein modulation upon CC2238/*α*ANP and TT2238/*α*ANP was subsequently obtained in independent experiments in HUVSMCs and also in CASMCs (number of experiments=6 for each cell line) by RT-PCR, based on SYBR Green methodology, and by western blotting of total proteins.

### Impact of NPR-C gene silencing on modulation of ApoE gene and protein upon CC2238/*α*ANP

HUVSMCs and CASMCs stimulation with CC2238/*α*ANP was repeated both in the presence and in the absence of NPR-C (number of experiments=6) to verify the role of this receptor in mediating the effects of CC2238/*α*ANP on ApoE gene and protein expression levels, according to our previous reports.^10, 11^

### Role of NPR-C-dependent activation of miRNA199a-3p and miRNA199a-5p in the modulation of ApoE by CC2238/*α*ANP. Involvement of Egr-1

To verify if a known epigenetic modulation of ApoE, dependent on miRNA199a-3p and 199a-5p,^14^ played a role in our experimental context, total RNA, extracted from both NPR-C not silenced and silenced HUVSMCs and CASMCs, was purified to isolate small RNAs by using a commercially available kit. For RT-PCR of DNAJA4, a known target of miRNA199a,^14^ a TaqMan gene expression assay was used. Next, based on previous knowledge that miRNA199a expression levels are modulated by Egr-1,^15^ we assessed the Egr-1 protein expression level upon either TT2238/*α*ANP or CC2238/*α*ANP by western blotting. We also better established the role of Egr-1 into the ApoE modulation under CC2238/*α*ANP with additional experiments. First, the known role of oxidative stress on Egr-1 stimulation^17^ was verified by exposing VSMCs to CC2238/*α*ANP both in the absence and in the presence of apocynin (number of experiments=3), following conditions previously described.^9^ Egr-1 expression levels were therefore assessed by western blotting. Subsequently, modulation of both miRNA199a-3p and miRNA199a-5p, of DNAJA4 and of ApoE was determined in Egr-1 silenced VSMCs exposed to CC2238/*α*ANP (number of experiments=4).

### Assessment of cleaved caspase-3, CREG, JNK, p38MAPK, Nf-*k*Bp65 and TGF*β* (Smad4) expression levels as markers of apoptosis, necrosis and inflammation upon CC2238/*α*ANP both in the presence and in the absence of NPR-C

To verify whether downregulation of ApoE expression in our experimental conditions was associated, as expected, to increased rate of apoptosis, necrosis and inflammation,^13^ total proteins were analyzed by using the following specific primary antibodies: anti-cleaved caspase-3, anti-CREG, anti-phosphoJNK, anti-JNK, anti-phospho-p38MAPK, anti-p38MAPK, anti-NF*κ*Bp65, anti-Smad4, anti-*β*-actin. The latter was used as the housekeeping protein. Following incubation with horseradish peroxidase-conjugated secondary antibody, signals were revealed as described below.

### Impact of exposure to recombinant ApoE on cell viability, apoptosis, necrosis and inflammation in both HUVSMCs and CASMCs in the presence of CC2238/*α*ANP

To test the ability of ApoE supplementation to rescue the harmful effects induced by CC2238/*α*ANP, and based on sequencing results of specific ApoE isoforms expressed in the two cell lines, HUVSMCs were exposed for 12 h to CC2238/*α*ANP and either recombinant ApoE2 or ApoE3 protein (10 *μ*g/ml; Sigma). CAMSCs were exposed to CC2238/*α*ANP and either recombinant ApoE3 or ApoE4 protein (10 *μ*g/ml; Sigma). Recombinant proteins for ApoE were added to VSMCs as previously described.^37^

Twenty-four hours after completion of exposure to CC2238/*α*ANP and the specific recombinant ApoE isoform, counting of apoptotic cells was performed by flow cytometry analysis (FACS) and total proteins were extracted for western blotting analysis of Nf-*κ*Bp65 and Smad4.

Experiments were performed in triplicate for each ApoE isoform.

### Cells culture

Commercially available HUVSMCs, purchased from ATCC (American Type Culture Collection; Manassas, VA, USA), were grown in Kaighn's F12 medium containing 2 mM l-glutamine, 1.5 g/l sodium bicarbonate, and supplemented with 0.1 mg/ml heparin, 0.03 mg/ml endothelial cell growth supplement, 10% fetal bovine serum. Commercially available CASMCs (purchased from Lonza, Walkersville, MD, USA) were grown in Smooth Muscle Growth Medium-2 (SmgM-2; Lonza). Four batches were used for each cell line to perform all experiments.

Cells were used within the fifth passage and at 70% confluence. For stimulation with either TT2238 or CC2238/*α*ANP, they were seeded in 24-well plates (8 × 10^4^ cells/well), cultured in their respective growth medium for 24 h, subsequently starved and stimulated with *α*ANP at a final concentration of 10^9^ mol/l for 12 h in the presence of 10% fetal calf serum. Control plates received *α*ANP-free medium.

### Total RNA extraction

Total RNA was obtained using Trizol reagent (Life Technologies, Carlsbad, CA, USA), subjected to DNAse I treatment (Qiagen, Venlo, Netherlands) and subsequently purified using RNeasy Mini Kit (Qiagen) according to the manufacturer's instructions. RNA integrity was assessed by denaturing agarose gel electrophoresis and its concentration was verified by using NanoDrop2000c UV-Vis spectrophotometer (Thermo Scientific, Waltham, MA USA).

### RT-PCR macroarray analysis

Three experiments were carried out with each stimulus and RNA was pooled for the macroarray analysis. Two micrograms of total RNA were used for cDNA synthesis using RT^2^ First Strand Kit (Qiagen). We carried out quantitative RT-PCR macroarray analysis using a Human Atherosclerosis RT^2^Profiler PCR Array purchased from Qiagen. Experiments were performed as previously described.^9^ Briefly, cDNA template was mixed with one of ready to use RT^2^ Real-Time SYBR Green PCR Master Mix (Qiagen). The chosen array contains a panel of 84 primer sets for pathway-focused genes (human atherosclerosis), five housekeeping genes and three RNA and PCR quality controls. The mixture was aliquoted into each well of the same plate containing predispensed gene-specific primer sets and PCR was performed on ViiA 7 Real-Time PCR System (Life Technologies). Experiments were performed in triplicate. Data analysis was carried out using the PCR Array Data Analysis Web Portal (http://www.superarray.-com/pcrarraydataanalysis.php) and ΔΔCt method.^38^ Results were expressed as relative levels of each gene mRNA under the effect of either CC2238 or TT2238/*α*ANP referred to the expression of this gene in unstimulated cells (that were chosen to represent 1x expression of each gene). Results were considered significant when mRNA expression was 5-fold higher or lower than that of unstimulated cells.

### Quantitative RT-PCR of ApoE, NPR-C, Egr-1 (SYBR Green methodology)

Two microliters of cDNA were mixed with 5 *μ*l of SYBR Green PCR master Mix (Life Technologies) and 0.5 *μ*l of specific primers: ApoE (forward 5′-GAGCAAGCGGTGGAGACAG-3′ and reverse 5′-CATCAGCGCCCTCAGTTCC-3′); NPR-C (forward 5′-GGAAGACATCGTGCGCAATA-3' and reverse 5'-GATGCTCCGGATGGTGTCA-3'); Egr-1 (forward 5′-ACCGCAGAGTCTTTTCCTGACA-3′ and reverse 5′-GGTGCAGGCTCCAGGGAAAA-3′).

*β*-Actin was used as the housekeeping gene (forward 5′-GCAAGAGATGGCCACGGCTG-3′ and reverse 5′-CCACAGGACTCCATGCCCAG-3′). RT-PCR was carried out on the ViiA 7 Real-Time PCR System with cycling conditions of 50 °C for 2 min, 95 °C for 10 min followed by 95 °C for 15 s and 60 °C for 60 s for a total of 40 cycles. Measurements were performed in triplicate in each assay. Results were expressed as relative levels of each gene mRNA as compared with control cells.

### Quantitative RT-PCR of miRNA199a-3p, miRNA199a-5p, DNAJA4 (TaqMan methodology)

Total RNA was purified to isolate small RNAs with a specific kit (miRNeasy Mini Kit; Qiagen) according to the manufacturer's instructions. For cDNA synthesis of miR199a-3p, miR199a-5p and control miRU87, 1 *μ*g of purified RNA was mixed with 3 *μ*l of NCode VILO miRNA cDNA Synthesis Kit (Life Technologies) and 1 × RT-primer (Life Technologies) in a total reaction volume of 20 *μ*l. Reaction was incubated at 16 °C for 30 min, 42 °C for 30 min and 85 °C for 5 min in a T-100 Thermal Cycler (Bio-Rad, Hercules, CA, USA). Then, 2 *μ*l of the RT reaction was added to 1 *μ*l of a specific TaqMan MicroRNA Assay 20 × (miR199a-3p, miR199a-5p, miRU87; Life Technologies) and 10 *μ*l of TaqMan Universal PCR Master Mix II with UNG (Life Technologies) in a 20 *μ*l final volume. RT-PCR was performed using the ViiA 7 Real-Time PCR System with cycling conditions of 95 °C for 10 min followed by 95 °C for 15 s and 60 °C for 60 s for a total of 40 cycles. Each TaqMan assay was run in triplicate. Results were expressed as relative levels of each miRNA under the effect of different treatments relative to unstimulated cells.

For RT-PCR of DNAJA4, 2 *μ*l of cDNA were mixed with 10 *μ*l of TaqMan Universal Master Mix II with UNG and 1 *μ*l of DNAJA4 TaqMan Gene Expression Assay (Life Technologies) in a 20 *μ*l final volume. *β*-Actin was used as the housekeeping gene. RT-PCR was performed using ViiA 7 Real-Time PCR System with cycling conditions of 50 °C for 2 min, 95 °C for 10 min followed by 95 °C for 15 s and 60 °C for 60 s for a total of 40 cycles. Each assay was run in triplicate. Results were expressed as mRNA levels under the effect of different treatments relative to unstimulated cells.

### Total proteins extraction and western blotting

Total proteins were extracted from cells 24 h after completion of either TT2238/*α*ANP or CC2238/*α*ANP exposure. Samples were lysed in 1 : 10 weight/volume in lysis buffer (50 mM NaCl, 100 mM Tris-HCl pH 7.4, 1 mM ethylenediaminetetracetic acid (EDTA), 1% SDS) supplemented with protease and phosphatase inhibitors (Sigma Aldrich, Saint Louis, MO, USA), incubated on ice for 15 min. and centrifuged at 13 000 r.p.m. for 15 min. Protein concentration was determined by DC Protein Assay Kit (Bio-Rad) following the manufacturer's instructions. Protein lysates were boiled at 95 °C for 5 min in the presence of gel loading buffer (Bio-Rad). Fifty micrograms of each sample were loaded on 10% SDS-polyacrylamide gel and run for 120 min at 100 V. Proteins were then transferred onto polyvinylidene difluoride membranes (PVDF; Bio-Rad) using the Trans-Blot Turbo Transfer System (Bio-Rad). Nonspecific binding sites were blocked at room temperature for 1 h in 5% bovine serum albumin in Tris-buffered saline buffer with 0.1% Tween 20 (Sigma) (TBS-T). Membranes were incubated at 4 °C overnight with the following primary antibodies:

anti-ApoE (1 : 1000; Millipore, Temecula, CA, USA), anti-Egr-1 (1 : 1000; Cell Signaling, Danvers, MA, USA), anti-phosphoJNK (1 : 200; Santa Cruz Laboratories, Santa Cruz, CA, USA), anti-JNK (1 : 200; Santa Cruz), anti-phospho-p38MAPK (1 : 200; Santa Cruz), anti-p38MAPK (1 : 200; Santa Cruz), anti-NF*κ*Bp65 (1 : 200; Santa Cruz), anti-cleaved caspase-3 (1 : 1000; Cell Signaling), anti-Smad4 (1:1000; Cell Signaling); anti-CREG (1:1000; R&D System, Minneapolis, MN, USA), anti-*β*-actin (1 : 1000; Santa Cruz).

Following three washes of 10 min in TBS-T, membranes were incubated for 1 h at room temperature with 1 : 5000 horseradish peroxidase-conjugated donkey anti-goat (for ApoE) or goat anti-mouse or goat anti-rabbit secondary antibodies diluted in TBS-T. After three washes of 10 min in TBS-T, signals were revealed with an enhanced chemiluminescence detection system (Luminata Crescendo; Millipore, Darmstadt, Germany) and the immunoreactivity of bands was visualized using ChemiDoc XRS+Imaging System (Bio-Rad). Protein bands were scanned and quantified densitometrically. They were finally normalized using β-actin levels.

### NPR-C and Egr-1 gene silencing

NPR-C and Egr-1 gene silencing was performed with specific siRNAs (Mission siRNA; Sigma) by following conditions previously described.^10, 11^ Twenty-four hours after transfection, both silenced and not silenced cells were exposed to CC2238/*α*ANP for 12 h and subsequently extracted for total RNA and total proteins. Efficiency of gene silencing was assessed by RT-PCR, following conditions described above.

### Role of oxidative stress in the Egr-1 induced increase by CC2238/*α*ANP

The known role of oxidative stress on Egr-1 stimulation ^17^ was verified by exposing VSMCs to CC2238/*α*ANP both in the absence and in the presence of apocynin, following conditions previously described.^9^ Egr-1 expression levels were therefore assessed by western blotting.

### Sequencing of apoE gene expressed in HUVSMCs and CASMCs

Genomic DNA was isolated from both HUVSMCs and CASMCs using a commercially available kit (Qiagen, Chicago, IL, USA). In order to identify the three allelic forms of human ApoE gene (allele ɛ2, ɛ3, ɛ4) PCR reactions were set up by using a specific pair of primers: forward 5′-CTCCCACTGTGCGACACC-3′, reverse 5′-CTGCTCCTTCACCTCGTCC-3′. DNA was first amplified by PCR with 40 cycles at an annealing temperature of 55 °C using a T-100 Thermal Cycler (Bio-Rad). Following purification of the PCR product (562 bp) with MinElute PCR purification kit (Qiagen), sequence reactions were prepared using the Big Dye Terminator v3.1 cycle sequencing Kit (Applied Biosystems, Carlsbad, CA, USA). Unincorporated dye terminators from sequencing reactions were removed using DyeEx 2.0 Spin kit (Qiagen). Purified fragments were electrophoresed on an ABI PRISM 3100 Genetic Analyzer (Applied Biosystems) according to the manufacturer's protocol. All sequencing results were compared with those present in the NCBI databases using BLAST to confirm the allelic variants.

### Flow-cytometry analysis

Twenty-four hours after completion of exposure to CC2238/*α*ANP and the specific recombinant ApoE isoform, counting of apoptotic cells was performed by FACS using Annexin V-Fitc and propidium iodide (PI) staining (ImmunoStep, Salamanca, Spain). For this purpose, cells were harvested by incubation with 1 ml of trypsin/EDTA (Lonza) for 3 min at 37 °C. Trypsinization was stopped by addition of medium and the suspension was centrifuged at 1200 r.p.m. for 5 min at 4 °C. Each pellet was washed with cold phosphate-buffered saline (PBS 1 × ). Then, tubes were vortexed thoroughly and centrifuged again as before. Cells were gently resuspended and vortexed in binding buffer at a concentration of 3 × 10^6^ cells/ml. Then, 100 *μ*l of cell suspension was added to 5 *μ*l of Annexin V and 10 *μ*l of PI. Samples were mixed for 15 min in the dark at 4 °C and 400 *μ*l of PBS 1x was added to the solution. Ten thousand cells were analyzed by FACS on a BD Accuri C6 flow cytometer (Biosciences, Erembodegem-Dorp, Belgium) to count apoptotic cells. Both negative control (untreated cells) and positive control (cells treated with hydrogen peroxide) were included in the analysis.

### Statistical analysis

Continuous variables are expressed as mean±S.D. Comparisons between two groups were performed using Student's *t-*test. When the analysis was adjusted for the multiplicity of compared groups, one-way ANOVA followed by Bonferroni *post hoc* test was performed. Normality of variables distribution was tested by the Kolmogorov–Smirnov test. GraphPad Software (San Diego, CA, USA; version 5.0) and SPSS statistical software (SPSS Inc., Chicago, IL, USA; version 12.0) were used for statistical analysis. *P*<0.05 was considered significant.

## Figures and Tables

**Table 1:** Atherosclerosis genes that are modulated by TT2238/αANP and CC2238/αANP *versus* control according to real time-PCR array

**Gene name**	**TT2238/***α***ANP** ***versus*** **Ctrl**	**CC2238/***α***ANP** ***versus*** **Ctrl**
*APOE*	38.48±18.57	−6.66±4.13
*BCL2*	9.37±3.84	−6.70±1.94
*BCL2A1*	16.62±39.98	−1.13±1
*BCL2L1*	24.24±14.69	133.34±79.02
*CCL2*	−736.48±426.11	−5514.23±2765.44
*CTGF*	−1.89±1	−12.14±15.01
*FGF2*	−40.18±19.21	0.29±3.78
*FN1*	−1855.62±888.82	−0.97±2.57
*ITGA2*	2.70±3.32	18.22±7.09
*ITGA5*	−0.95±5.45	−20.05±7.52
*MMP3*	10.57±7.79	21.57±14.94
*NOS3*	−24.76±1	−2.51±1
*PDGFB*	8.07±4.96	0.04±2.52
*PPARD*	−6.05±1.87	−0.93±1.94
*PPARG*	−7.16±1.36	−4,57±0,33
*SERPINE1*	−2.61±2.55	−5.84±0,67
*SOD1*	−4.77±7.16	1.22±3.20
*TNC*	−109.95±63.82	−1.80±2.52
*TNF*	6.04±5.03	−2.59±6.53

Number of independent experiments=3.

*BCL2*, B-cell lymphoma 2; *CCL2*, chemokine ligand 2; *CTGF*, connective tissue growth factor; *FGF2*, fibroblast growth factor 2; *FN1*, fibronectin 1; *ITGA2*, integrin alpha 2; *ITGA5*, integrin alpha 5; *MMP3*, matrix metallopeptidase 3; *NOS3*, nitric oxide synthase 3; *PDGFB*, platelet-derived growth factor subunit B; *PPARD*, *PPARG*, peroxisome proiliferator-activated receptor delta, gamma; *SERPINE1*, serpin peptidase inhibitor; *SOD1*, superoxide dismutase 1; *TNF*, tumor necrosis factor.

## Abbreviations

Akt: protein kinase B
ApoE: apolipoprotein E
ANP: atrial natriuretic peptide
TT2238/*α*ANP: wild-type atrial natriuretic peptide at pos. 2238
CC2238/*α*ANP: mutant atrial natriuretic peptide at pos. 2238
cAMP: cyclic adenosine monophosphate
CREG: cellular repressor of E1A-stimulated gene
CREB: cAMP responsive element binding protein
DNAJA4: DnaJ (Hsp40) homolog
Egr-1: early growth response protein-1
eNOS: endothelial nitric oxide synthase
EDTA: ethylenediaminetetracetic acid
FOXO4: forkhead box O4
FACS: flow cytometry analysis
JNK: c-Jun N-terminal kinase
miRNA: microRNA
NADPH: nicotinamide adenine dinucleotide phosphate-oxidase
Nf-*κB*: nuclear factor kappa-light chain-enhancer of activated B cells
NPR-C: type C natriuretic peptide receptor
NPR-A: type A natriuretic peptide receptor
PKA: protein kinase A
ROS: reactive oxygen species
RT-PCR: real time-polymerase chain reaction
Smad4: mothers against decapentaplegic Homolog 4
TGF: tumor growth factor
VSMC: vascular smooth muscle cell
HUVSMC: human umbilical vein smooth muscle cell
CASMC: coronary artery smooth muscle cell
