# Supplementary material for: C2238/αANP modulates apolipoprotein E through Egr-1/miR199a in vascular smooth muscle cells in vitro
Source: Cell Death Dis. 2015 Dec 31;6(12):e2033–. doi: 10.1038/cddis.2015.370 (PMC4720902; doi:10.1038/cddis.2015.370)

**SUPPLEMENTARY INFORMATION**

# C2238/αANP modulates Apolipoprotein E through Egr-1/miR199a in vascular smooth muscle cells in vitro

Rosita Stanzione[1*], Sebastiano Sciarretta[1,2*], Simona Marchitti[1], Franca Bianchi[1], Sara Di Castro[1], Stefania Scarpino[3], Maria Cotugno[1], Giacomo Frati[1,2], Massimo Volpe[1,3], Speranza Rubattu[1,3]

[1]IRCCS Neuromed, Pozzilli; [2]Department of Medical-Surgical Sciences and Biotechnologies, Sapienza University of Rome, Latina; [3]Department of Clinical and Molecular Medicine, School of Medicine and Psychology, Sapienza University of Rome, Ospedale S. Andrea, Rome; Italy

*these authors contributed equally to this work

Running title: C2238/αANP and Apolipoprotein E

Correspondence to:

Speranza Rubattu, MD.
Clinical and Molecular Medicine Department,
School of Medicine and Psychology
Sapienza University, S.Andrea Hospital, Rome;
IRCCS Neuromed, Pozzilli (Is), Italy
e-mail: rubattu.speranza@neuromed.it
Tel. 0039 06 33775979; Fax 0039 06 33775061

## Supplementary Figure legends

### Supplementary Figure 1

***Role of oxidative stress on Egr-1 stimulation by CC2238/αANP***

Cells exposed to CC2238/αANP showed significant increase of Egr-1 expression levels, which was abolished in the presence of apocynin. Number of independent experiments=3.

CC2238/αANP = variant αANP; Egr-1= early growth response protein-1; CTR= control

### Supplementary Figure 2

***Modulation of proteins involved in apoptosis, necrosis and inflammation by CC2238/αANP in HUVSMCs***

Panels A-F. Representative western blots of cleaved-caspase-3, CREG, JNK, p38MAPK, Nf-kBp65, Smad4 under CC2238/αANP both in the presence and in the absence of NPR-C. Corresponding densitometric analysis is shown below each blot.

Data are expressed as mean ± SD. Number of independent experiments=6.

** $p<0.0001$ for CC2238/αANP vs all other points.

CC2238/αANP = variant αANP; siRNA1=NPRC gene silencer 1; siRNA2=NPRC gene silencer 2; CREG=cellular repressor of E1A-stimulated gene; JNK=c-Jun N-terminal kinase; CTR=control.

### Supplementary Figure 3

***Modulation of proteins involved in apoptosis, necrosis and inflammation by CC2238/αANP in CAMSCs***

Panels A-F. Representative western blots of cleaved-caspase-3, CREG, JNK, MAPKp38, Nf-kB, Smad4 under CC2238/αANP both in the presence and in the absence of NPR-C. Corresponding densitometric analysis is shown below each blot.

Data are expressed as mean ± SD. Number of independent experiments=6.

** $p<0.0001$ for CC2238/αANP vs all other points.

CC2238/αANP = variant αANP; siRNA1=NPRC gene silencer 1; siRNA2=NPRC gene silencer 2; CREG=cellular repressor of E1A-stimulated gene; JNK=c-Jun N-terminal kinase; CTR= control.

**Supplementary Figure 4**

Results of ApoE gene sequencing in both HUVSMCs (panel A) and CASMCs (panel B).

HUVSMCs express both ApoE2 (Cys112/Cys158) and ApoE3 (Cys112/Arg158) isoforms.

CASMCs express both ApoE3 (Cys112/Arg158) and ApoE4 (Arg112/Arg158) isoforms.

Cys=cysteine; Arg=arginine

Supplementary Figure 1

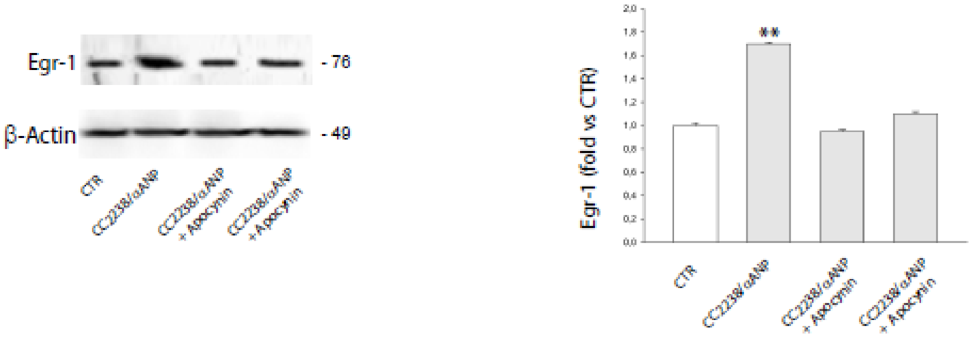

Supplementary Figure 2

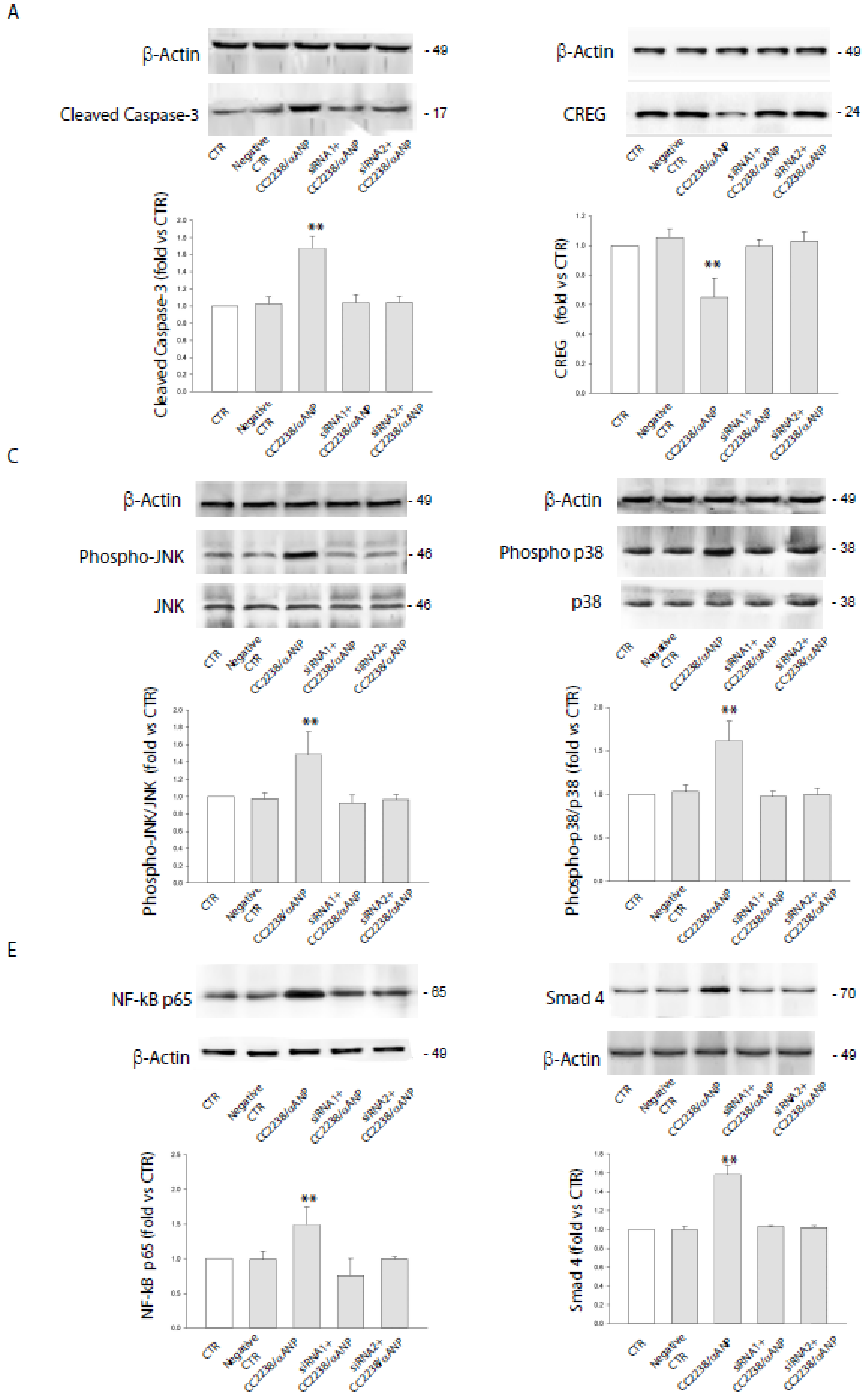

Supplementary Figure 3

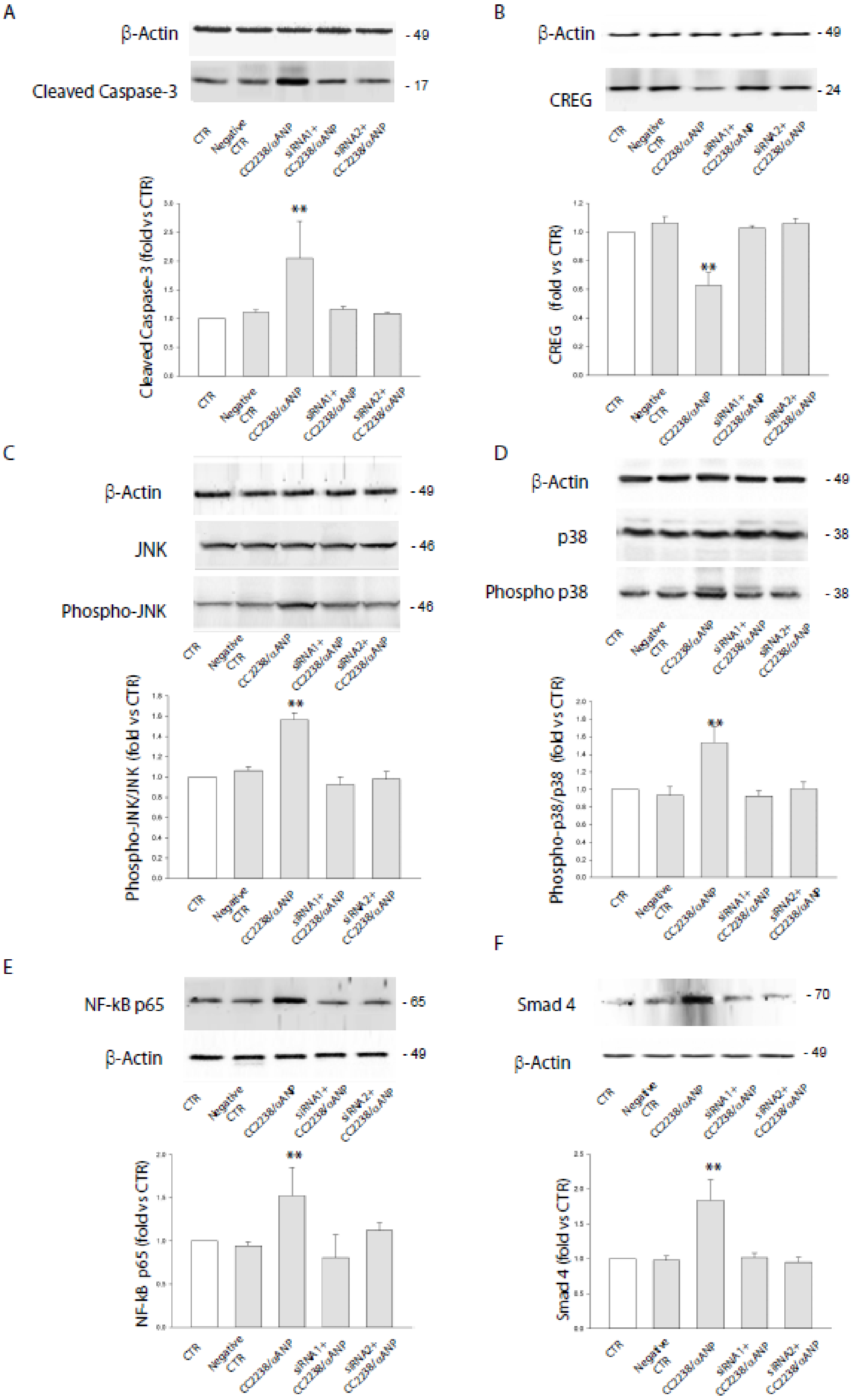

Supplementary Figure 4

A

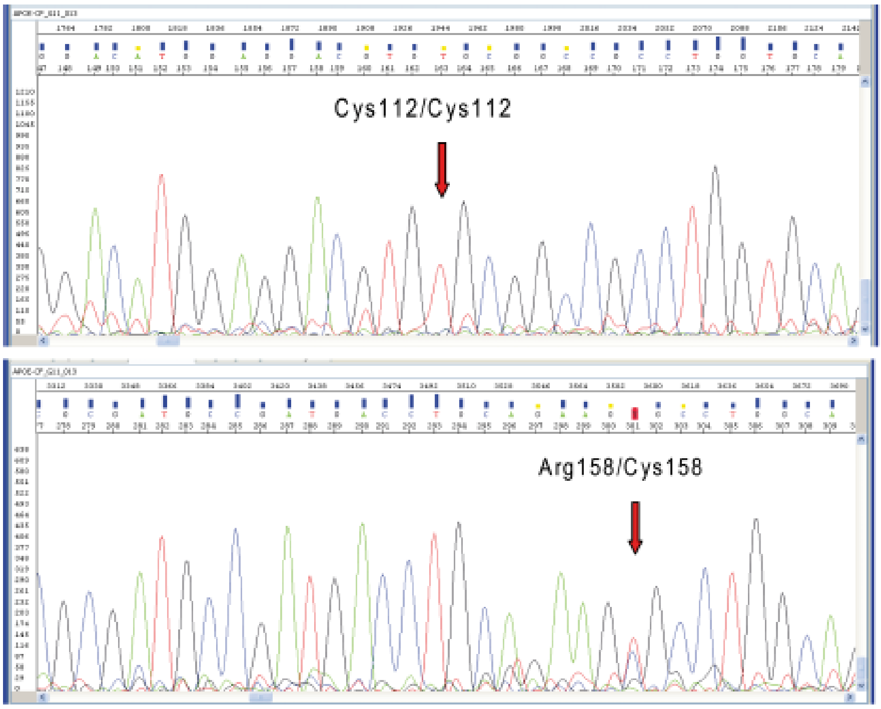


B

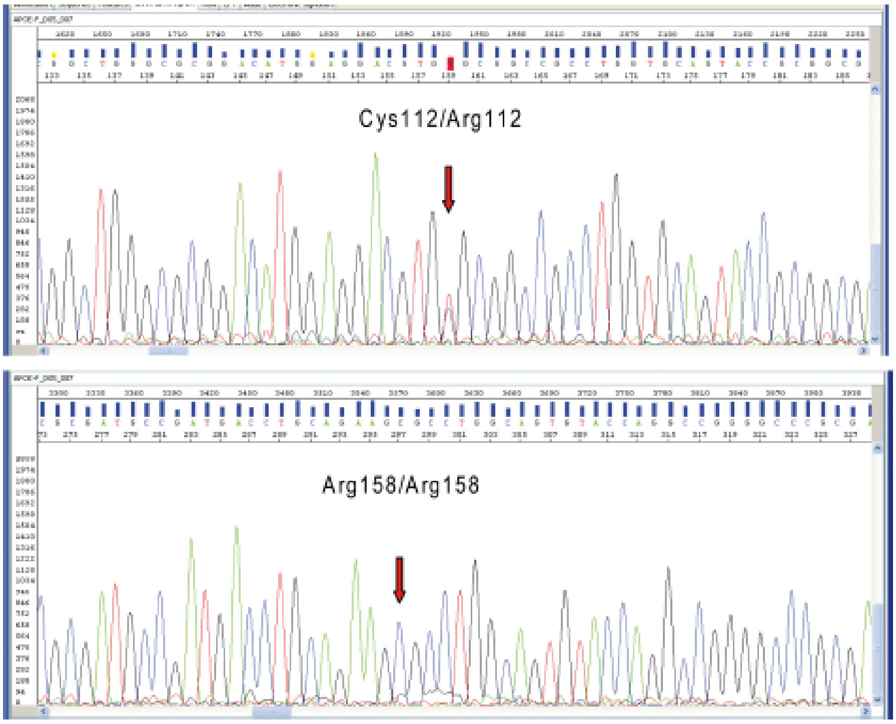

Supplement: Supplementary Information [file cddis2015370x1.pdf]
